# Uranium in cobalt-hydroxide exports from the Democratic Republic of the Congo

**DOI:** 10.1038/s41467-026-75910-z

**Published:** 2026-07-30

**Authors:** Ryan A. Manzuk, Sébastien Philippe

**Affiliations:** 1 Program on Science and Global Security, Princeton University, Princeton, NJ USA; 2 Department of Nuclear Engineering and Engineering Physics, University of Wisconsin-Madison, Madison, WI USA

**Keywords:** Economic geology, Energy science and technology, Energy and society, Energy and society

## Abstract

The Democratic Republic of Congo (DRC)’s Copperbelt is the world’s leading source of cobalt, a metal essential to the energy transition. In this region, uranium co-occurs with oxidized cobalt ores. Despite no officially reported uranium production, we show that uranium is extracted, concentrated, and exported via the DRC cobalt supply chain. This conclusion follows from co-mineralization, similar behavior of cobalt and uranium in hydrometallurgical circuits, and the partitioning of uranium into crude cobalt hydroxide, the export form for over 95% of DRC production. We combine a first-order carryover model with geological mapping, mineralization and geochemical data, as well as mine-level trade records (2000–2024) to quantify these flows. We estimate 2,000–5,000 tonnes of natural uranium were exported embedded in cobalt-hydroxide shipments. Less than 10% of this material has been publicly declared and placed under international safeguards. In addition, 1,000–4,000 tonnes were likely discarded to tailings in easily-mobilized forms, posing environmental and health risks. Together, these findings identify a substantial gap in nuclear accountancy and environmental oversight within a critical mineral supply chain.

## Abstract (web summary)

Uranium co-occurs with ores in the DRC, a major source of global cobalt supply. Known methods to remove uranium before export are not widely adopted. This paper finds that this supply chain likely has exported 2,000- 5,000 t of unreported uranium.

## Introduction

The Democratic Republic of Congo (DRC) was the original source of uranium ore for the first U.S. nuclear weapons built in 1945^1^. Today, the Shinkolobwe uranium deposit, exploited during the Manhattan Project, is officially closed^2,3^, and the country reports no uranium exports, including to the International Atomic Energy Agency (IAEA)^4^. The uranium-rich south remains intensely mined for other resources, especially copper and cobalt^5^, making the DRC the world’s second-largest copper producer and the leading source of mined cobalt in 2024^6,7^. These activities create pathways for incidental co-extraction, or deliberate recovery of uranium and pose occupational radiation-exposure risks.

**Fig. 1:**
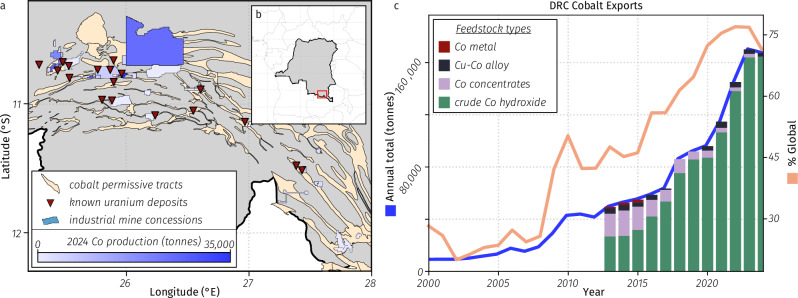
Cobalt mining in the uranium-rich Copperbelt, Democratic Republic of Congo (DRC). **a** Over two-thirds of the world’s cobalt supply is mined in the DRC’s southern Copperbelt. Economic-grade cobalt deposits occur mainly within outcrops of Roan Group sediments^27^. These rocks also contain elevated uranium levels, both as high background content^8^ and as discrete ore bodies often proximal to productive cobalt mine concessions. **b** Inset: location of the Copperbelt region (red outline) within Africa and the DRC. **c** Over the past 25 years, the DRC has become the preeminent global source of cobalt, rising from about 30% at the turn of the century to over 75% in recent years. Although a 2006 presidential decree required cobalt to be processed and exported as metal^31^, the overwhelming majority is exported as crude cobalt hydroxide. Administrative boundaries in panels (**a**, **b**) from the Global Administrative Areas database (GADM; https://gadm.org). Annual DRC export totals from refs. ^16^ and ^25^; global production shares from refs. ^16^ and ^24^; feedstock types from ref. ^16^. Panels (**a**, **b**) produced with GeoPandas^84^ and Matplotlib^85^. Panel (**c**) produced with Matplotlib^85^.

In the DRC Copperbelt, uranium is not confined to discrete deposits such as Shinkolobwe, but is widespread in ores throughout the region’s characteristic outcrops of metalliferous rocks (Fig. 1a). The local mining industry, active for more than a century, has long had to contend with this uranium presence^8–10^. Elevated radioactivity has been documented at industrial copper sites^11^ and in ore shipments^12^, and artisanal miners have shown signs of internal uranium contamination^13^.

Recently, rising demand from lithium-ion battery manufacturers, especially for electric vehicles^14,15^, has pushed DRC cobalt output to over 150,000 tonnes annually in recent years, accounting for two-thirds of global supply^16^ (Fig. 1b). This sustained surge heightens uranium-related risks because cobalt and uranium are tightly coupled along the supply chain from extraction through export. In the DRC, the principal cobalt-bearing mineral, heterogenite (CoO(OH)), is an oxyhydroxide that can uptake oxidized uranium species and accumulate high uranium concentrations up to 3% by weight^17–19^. Unlike copper, cobalt is rarely refined domestically to metal or high-purity salts, and is exported primarily as crude cobalt hydroxide^16,20^ (Fig. 1b). Data for DRC fraction of global cobalt production and export types over time can be found in our Supplementary Data 2.

In hydrometallurgical circuits used to concentrate cobalt for export, cobalt and uranium exhibit similar chemical behaviors. Without dedicated removal steps, uranium remains in the cobalt stream and partitions into the exported cobalt-hydroxide product^20,21^. At least one documented case illustrates this coupling: from 2010 to 2017, the Kokkola Chemicals facility in Finland declared and sold uranium recovered during purification of DRC-origin cobalt products^22^, despite the absence of any declared uranium exports from the DRC in that period^4^. This case, originating from a single facility, remains one of the only publicly documented instances of uranium entering the international supply chain via DRC cobalt production and being placed under international safeguards, even though uranium is expected to be prevalent throughout the industry.

Taken together, these observations motivate a systematic assessment of uranium flows from DRC cobalt mines and refineries. Large cobalt production volumes^16^, combined with the documented co-occurrence of cobalt and uranium^17,18^, imply that consequential quantities of uranium may be involved. Moreover, refineries must make capital investments in dedicated uranium-removal systems^21^, and under-regulation typical of rapidly-expanding extractive sectors^23^ creates uncertainty as to whether this uranium is properly accounted for and disposed of in an environmentally-safe manner.

Here, we estimate the total uranium produced in 2000–2024 as a byproduct of cobalt mining and processing, including uranium exported from the DRC (embedded in cobalt-hydroxide shipments) and uranium discarded in tailings near mining and refining sites. We combine historical cobalt production, trade and export data from the literature and commodity reports^15,16,24,25^, with geochemical datasets^26^, as well as maps of uranium and cobalt occurrences in the DRC^8,19,27–29^. We use these data in concert to develop models estimating geological uranium prevalence across the Copperbelt and its carryover during cobalt-hydroxide production.

We find that approximately 2000–5000 tonnes of uranium were likely shipped out of the DRC alongside cobalt hydroxide, despite a 2002 mining-code amendment designating uranium as a restricted material^30^. We also estimate that a comparable quantity was extracted and incidentally consigned to tailings, which may present remobilization risks. These findings have implications for worker health and safety across the supply chain, for communities near mining and refining sites, as well as for nuclear non-proliferation and safeguards.

## Results

### Uranium extraction as a byproduct of cobalt

Uranium accompanies cobalt from ore through export because Co–U co-mineralization and similar aqueous chemistry keep uranium with cobalt in hydrometallurgical circuits. Absent targeted removal, most uranium partitions into the cobalt-hydroxide product.

These pathways reflect vulnerabilities typical of a surging extractive industry. As in Chile’s Atacama Desert, where cobalt and lithium extraction has driven water stress^23^, regulatory and infrastructure capacity in the DRC has not kept pace with rapid growth. The clearest illustration of production outpacing regulation in the DRC is the export form. In 2006, the government required cobalt to be exported as refined metal to capture value addition^31^. Yet inadequate electricity supplies make large-scale electrowinning impracticable^32^, and lacking enforcement^31^ has led to recent exports being majorly non-metallic forms. The trend in feedstock export type has continued to shift toward less-processed crude cobalt hydroxide (Fig. 1b). This contrasts with copper, which is less energy-intensive to isolate and electrowin^33^ and is exported as metal from the DRC^34^.

Beyond illustrating enforcement gaps that elevate uranium risk along the supply chain, this example also explains why DRC cobalt exports can carry substantial uranium. With little in-country value addition, refineries produce the least expensive concentrate for shipment, rather than refined metal^31,35^. Cobalt hydroxide, typically 50–100-fold richer than ore, does not selectively exclude impurities at precipitation^20^. Consequently, unless unwanted elements are rejected during physical upgrade or early hydrometallurgical steps, they co-precipitate and travel with the cobalt product. Below, we outline the key steps in producing cobalt-hydroxide concentrate to identify where uranium mined alongside copper and cobalt is most likely to be retained, diverted to tailings, or deliberately removed.

In the DRC, copper and cobalt are mined from both oxide and sulfide ores. Here, we focus on oxide ores, which dominate DRC cobalt exports and exhibit the strongest U–Co coupling during processing^20^. Implications of sulfide-ore processing are addressed later when we report export and tailings totals.

**Fig. 2:**
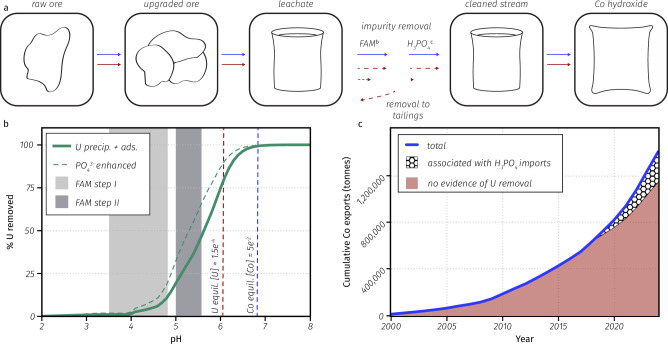
Uranium carryover pathways in the Democratic Republic of Congo cobalt supply chain. **a** Throughout cobalt hydroxide production chemistry, uranium is expected to follow cobalt in equal proportion. Incidental removal can occur during iron–aluminum–manganese (FAM) precipitation; targeted removal requires a post-FAM step (e.g., phosphoric-acid addition) to precipitate uranium selectively. **b** Uranium removal via precipitation/adsorption increases with pH but only approaches completeness near pH 7, where cobalt also precipitates and is lost. In our first-order model, a single low-pH FAM step removes *<*10% U; adding a second step at pH *> *5 yields ∼50% U removal at most. **c** Cumulative cobalt exports partitioned by evidence of targeted uranium removal (phosphoric-acid addition) show that nearly 1,200,000 tonnes of exports lack evidence of bulk uranium removal. Panels (**b**, **c**) produced with Matplotlib^85^.

*Ore upgrade and leach*—Ore upgrade (Fig. 2a) involves crushing rocks into constituent mineral grains, and using physical methods (e.g., gravity separation) to concentrate minerals of interest and discard gangue^36^. Typical DRC refineries achieve roughly a threefold increase in Cu–Co grades at this stage^20,36^. Because uranium and cobalt occur within the same mineral in oxidized ores, uranium is not expected to be rejected at this stage. Upgraded ore is then leached in sulfuric acid (Fig. 2a). At the typical leach pH of ∼1.5, uranyl species are highly soluble^20,21,37,38^, so uranium continues to track cobalt with negligible loss. Copper is recovered first from the leach liquor by targeted solvent extraction, and the remaining bleed stream is directed to cobalt concentration^20,21,33,35^.

*Impurity removal through pH manipulation*—Prior to cobalt-hydroxide precipitation, impurities are removed by increasing pH^20,33,35^. In moderately oxidizing bleed streams, cobalt precipitates near pH 6.8^37,39^, whereas bulk impurities, including iron, aluminum, and manganese (FAM), precipitate at lower pH (Fig. 2b). Refineries therefore add lime to raise pH modestly from leach conditions, precipitating FAM oxides/hydroxides to tailings while keeping most cobalt in solution^20,33,35^. Uranium, present at trace concentrations in the bleed stream^21^, has an equilibrium precipitation pH of ∼6.1, close to cobalt’s (Fig. 2b); accordingly, pH manipulation alone is an inefficient mechanism for uranium removal.

During FAM removal, some uranium can be scavenged from solution by adsorption onto metal hydroxides, particularly iron^40^. Adsorption increases with pH^40^ and may be enhanced by phosphate ions introduced from dissolved phosphate minerals^41^. Uranium removed by precipitation or adsorption reports to tailings predominantly as mobile uranyl or hydroxide species that can readily be dissolved or re-leached^42^. We refer to this fraction as *unstable tailings*.

To quantify these processes, we develop a first-order, pH-dependent model for uranium in the cobalt stream that includes both precipitation and adsorption (Fig. 2b). The model is intentionally not plant-specific. Flowsheets and ore feeds vary widely across Copperbelt operations^26^, and large hydrometallurgical circuits can deviate from laboratory equilibria, with redox states not consistently maintained^43^. Accordingly, this model is a first-order approximation of the average behavior of uranium under pH adjustment, which we interrogate under alternative processing scenarios.

In a first scenario consistent with minimal impurity control (i.e., maximum uranium export), refineries perform a single FAM precipitation step near pH 4, removing *<*10% of uranium prior to export (Fig. 2b). This pathway is likely as low-pH FAM is the fastest, lowest-cost route to a marketable cobalt hydroxide^20,33^. Market behavior has favored ramping output and depressing prices^31,44^, and a survey of DRC plants reports that operations shipping crude cobalt hydroxide typically implement only this single, low-pH step^20^.

By contrast, if refineries add a second FAM precipitation step at pH > 5 (e.g., ∼5.5)^33,35^, roughly 50% of uranium is removed and diverted to unstable tailings. However, we expect this scenario to be less common given the limited economic incentive for additional processing^16,31^, and the fact that precipitates formed during higher pH impurity removal are often re-leached^20^, which would return uranium back into the processing line. We therefore treat this case as a plausible upper bound for incidental uranium removal. Operating substantially above pH 5–5.5 incurs substantial cobalt and copper losses^45^, further suggesting that this scenario represents an unlikely, near-maximum estimate of uranium deportment to unstable tailings.

*Deliberate uranium removal* – The literature indicates that most uranium remains in solution through FAM and is carried into cobalt hydroxide unless specific post-FAM measures are applied^20,21^. Two targeted approaches recently have been proposed to lower uranium contents in cobalt hydroxide to international shipping specifications: (a) ion-exchange (IX) resin beds and (b) phosphoric-acid addition after FAM to precipitate uranium as a stable autunite mineral^20,21^. Both solutions require sustained procurement of reagents/equipment from outside the DRC.

**Fig. 3:**
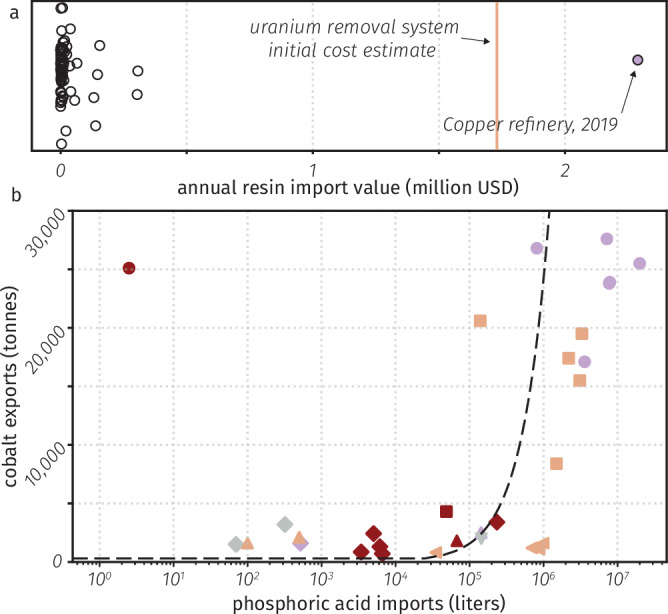
Limited adoption of targeted uranium removal in the DRC based on import data. **a** Ion-exchange resin imports rarely reach the capital requirement (~1,700,000 USD) for large-scale uranium removal. The only large purchase is associated with a copper-only facility and is unrelated to cobalt-stream uranium removal. **b** Of the 31 mines in our sample, only 11 show any phosphoric-acid imports, and only three consistently meet a first-order benchmark of ∼1,000,000 L per 25,000 t Co exported (black dashed line). Figure produced with Matplotlib^85^.

We gauge the implementation of these approaches across the industry by analyzing mining operation-tagged import records for relevant chemicals. We find no evidence of IX-resin deployment (Fig. 3a). Further, only three operations consistently import sufficient phosphoric acid to treat their exports, with few others doing so in 2023–2024 (Fig. 3b). By production share, only ∼20% of cumulative cobalt exports show evidence of targeted uranium removal to *stable tailings* by 2024 (Fig. 2c). Reducing uranium concentrations in cobalt exports below the 75 ppm international shipping standard requires deliberate implementation of phosphoric acid precipitation or ion-exchange removal^21^. The limited adoption documented here suggests that consistent compliance with this threshold is unlikely across the industry. Data for both IX resin and phosphoric acid imports to the DRC can be found in Supplementary Data 3 and Supplementary Data 4, respectively.

Although phosphate-bearing minerals (e.g., pseudomalachite) co-occur with heterogenite and can contribute phosphate ions during leach^20,34^, endogenous phosphate is unlikely to achieve substantial uranium removal. During FAM, Fe and Al preferentially consume phosphate^21^, so its main effect is to enhance uranium adsorption onto metal hydroxides^41^ rather than to drive bulk precipitation (Fig. 2b). At least one mine is mentioned in the literature as having its uranium naturally removed from cobalt streams and stably precipitated in tailings^20^, but its environmental and social impact assessment from 2021 mentions at least some addition of phosphoric acid being necessary to lower uranium levels^46^. Based upon available evidence, pseudomalachite-driven uranium removal likely is a rare phenomenon, specific to some phosphate-rich portions of exceptional deposits.

### Modeling uranium prevalence in cobalt ores

Quantifying uranium flows begins with constraining how much uranium enters the system at the mine. We estimate $\frac{{{\rm{U}}}\%}{{{\rm{Co}}}\%}$ ratios by integrating published cobalt grades with geologic mapping and uranium measurements, capturing both region-wide background and local enrichments near uranium deposits. We then apply Eq. (1) to propagate these ratios to mine-level exports. Cobalt exports from DRC operations are well-constrained over our study period^1,25^, and the industry commonly assumes ∼10% cobalt losses from extraction to export^1^. Accordingly, the uranium co-extracted with cobalt can be expressed as

1 $${{{\rm{U}}}}_{{{\rm{extracted}}}}=\frac{{{{\rm{Co}}}}_{{{\rm{exported}}}}}{0.9}\cdot \frac{{{{\rm{U}}}\%}_{{{\rm{ore}}}}}{{{{\rm{Co}}}\%}_{{{\rm{ore}}}}},$$

where U%_ore_ and Co%_ore_ denote the uranium and cobalt ore grades, respectively.

We compile cobalt ore grades for industrial deposits from published compilations^27^ and from available public company filings^47^. By contrast, uranium grades for industrial ores are rarely public. We therefore synthesize available uranium measurements from the region and geological descriptions of uranium prevalence and mobility in cobalt-bearing sediments to construct plausible scenarios for uranium grades in Copperbelt mines^8,13,19,28,48–53^. Two features must be constrained: (1) a region-wide elevated background applicable across the Copperbelt, and (2) locally very high grades proximal to known uranium deposits.

**Fig. 4:**
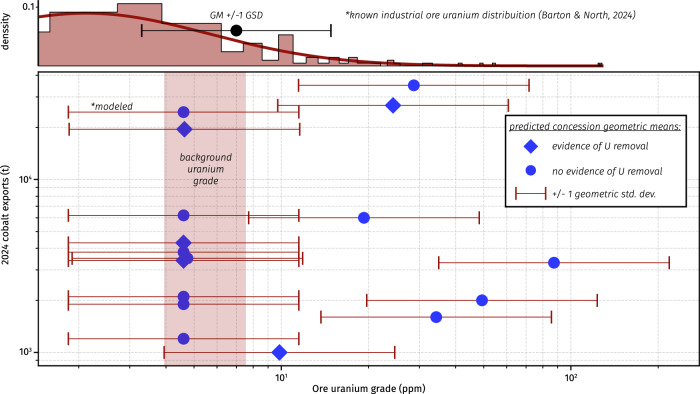
Known and predicted uranium ore grade distributions. The 134 samples^26^ follow a log-normal distribution (*R*^2^ = 0.91) with geometric mean (GM) 6.97 ppm and geometric standard deviation (GSD) 2.57. For the modeled intermediate-mobility scenario, arithmetic means are converted to GMs, consistent with log-normal geochemical grades. We assign this mine a background value of 7 ppm in the model, producing a small discrepancy with the measured GM and underscoring that our estimates are conservative. Uranium-export totals in this scenario will not be driven by a few high-grade, high-throughput mines: in 2024, 55% of cobalt production came from mines with background-level uranium grades. Evidence for phosphoric-acid uranium removal is diffuse, appearing at operations with background as well as slightly elevated grades. Figure produced with Matplotlib^85^.

*Uranium background across the Copperbelt* – Uranium averages ∼2.7 ppm in Earth’s crust^54^. At that bulk grade, Eq. (1) implies *>*100 t U would have been co-extracted with the 160,000 t Co exported in 2024 (Fig. 1b), assuming an overall ore grade of 0.4% Co^55^. Multiple studies show Copperbelt ores typically exceed this background^8^. At the lower end, a regional radiometric survey reports 4.2–4.4 ppm U, averaging across all lithologies, including poor U hosts^53^. At the upper end, artisanal-ore measurements of 28, 38, and 83 ppm U^13^, which reflect higher heterogenite content than industrial ores^56^. These grades normalize to a mean of ∼7.0 ppm when adjusted to an industrial 0.4% Co grade using the samples’ reported Co contents. Additionally, 134 geometallurgical measurements from one industrial mine^26^ provide a direct reference for a productive cobalt mine located far from any known uranium deposits (SI Fig. 1a). The uranium concentrations are log-normally distributed, with a geometric mean of 7.0 ppm and a geometric standard deviation of 2.6, corresponding to a one-sigma range of 2.7–18 ppm (Fig. 4). Across multiple studies, background scenarios consistently show values between approximately 4 and 17 ppm. Assuming a log-normal distribution, we therefore adopt 7 ppm as a conservative background uranium concentration for cobalt ores in the Copperbelt.

*Higher uranium grades along faults*—Neoproterozoic-to-Cambrian regional tectonic events 650 and 530 million years ago (Ma) concentrated uranium into discrete, high-grade deposits along faults in the Copperbelt^19,28^. We exclude direct (often artisanal) exploitation of these uraninite mineralizations, such as Shinkolobwe^3^, from our estimates. However, we account for elevated uranium in adjacent cobalt-ore occurrences due to more recent mobilization and redeposition.

**Fig. 5:**
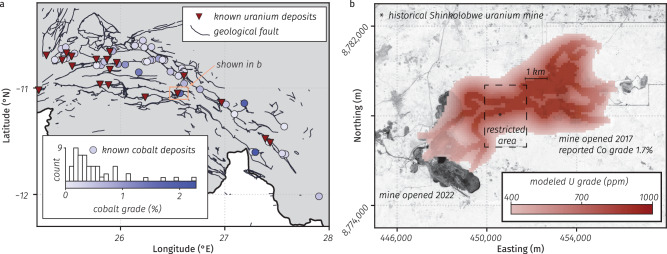
Uranium mobilization along faults and the Shinkolobwe case. **a** The sediments that are most likely to contain cobalt and uranium in the Democratic Republic of Congo (DRC) have been severely broken up and displaced by tectonic events^27^. As a result, these metalliferous deposits preferentially occur along or nearby fault lines^49^. Economically-viable cobalt exploitations have a typical grade of 0.4%^20^. Administrative boundaries from the Global Administrative Areas database (GADM; https://gadm.org) **b** Uranium grades in economic cobalt deposits are rarely published. To fill this gap, we model potential uranium mobility with preferential transport along faults. In the maximum-mobility scenario, elevated U extends to mines permitted within 1 km of the restricted Shinkolobwe uranium area; at least one such mine reports a high cobalt grade (1.7%), consistent with metals mobilized from Shinkolobwe and implying cogenic uranium may also be elevated. Background is an inverted grayscale image with 3 meter-per-pixel resolution captured by a Planet Dove satellite on 12 December 2024 (Imagery © 2024 Planet Labs Inc.; Planet Team (2026). *Planet Application Program Interface: In Space for Life on Earth*. San Francisco, CA. https://api.planet.com). Panel (**a**) produced with GeoPandas (https://geopandas.org) and Matplotlib^85^. Panel (**b**) produced with GeoPandas^84^, Matplotlib^85^, and Rasterio (https://rasterio.readthedocs.io).

Beginning 80 Ma^57^, and intensifying between 10 and 5 Ma due to regional uplift, meteoric weathering in oxidizing conditions mobilized buried metals (Co, Mn, U)^50^, and redeposited them preferentially along faults^19,48,58^ (Fig. 5a). In the case of uranium and cobalt, this oxidative mobilization creates proper conditions for co-precipitation as oxidized uranium may achieve grades up to 3% by weight in heterogenite crystals^17–19^. Adsorption of uranyl groups onto the highly specific surfaces^19^ of heterogenite is thought to be the mechanism for this association, even in extremely high-grade uranium examples^18,19^, but uranyl substitutions into the lattice or inclusion of sub-micronic uranium oxides also are possible mechanisms^17,19^.

To capture how this geology influences uranium levels, we implement a mobilization model that generates scenarios of elevated U in cobalt ores near uranium deposits. We seed ores immediately adjacent to deposits at 1000 ppm U, which is a conservative value obtained by diluting the 2–4% U measured in heterogenite crystals within concentrated deposits^19^ to typical bulk-ore grades. Bulk-rock values exceeding 1000 ppm, ranging to 10,000 ppm, are also consistently reported in these deposits^53,59^, and so our seed ores are a very conservative underestimate. Uranium then propagates outward in all directions, with preferential transport along mapped faults (see *“Methods”: Model of uranium mobility*). Because the model is iterative, we analyze snapshots at increasing propagation to compare low-, intermediate-, and high-mobility regimes and their implications for uranium co-extraction with cobalt.

The historical Shinkolobwe uranium mine is co-enriched in cobalt and nickel^19,51,52^. Combined with evidence that Co and U are cogenic and co-mobilized^52^, the local faulted geology implies that elevated uranium should persist in fault-connected deposits near the mine. Consistent with this expectation, our maximum-mobility scenario yields a near-1000 ppm U isopleth extending up to 3 km from Shinkolobwe along a dense fault network (Fig. 5b). This result is consistent with the anomalously high cobalt grade (1.7%) reported at a mine 1 km east of the restricted uranium mining area^27^. The eastern mine (opened in 2017) and another to the west (opened in 2022) have not yet been reported as major cobalt producers^16^. If output increases, our model suggests both sites would be expected to co-produce substantial quantities of uranium.

### Total uranium in exports and tailings

**Fig. 6:**
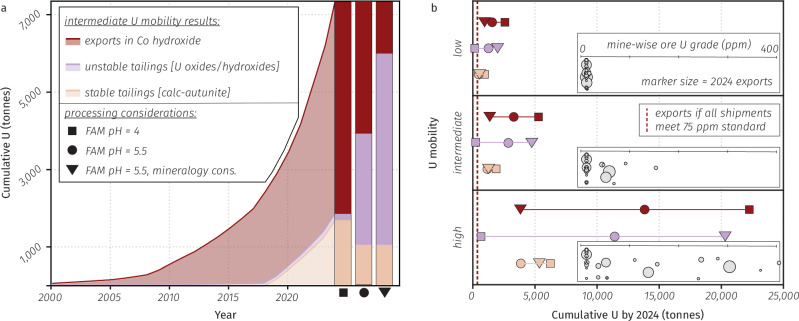
Modeled uranium in exports and tailings from the DRC cobalt industry (2000–2024). **a** Uranium mobility governs total uranium extracted, while processing determines its partitioning between exports and tailings. Under the *intermediate* mobility scenario, *>*7000 t U are extracted over the study period, with a sharp rise after 2017 as Co exports exceed 80,000 t yr^−1^. Given little evidence of deliberate uranium removal via phosphoric acid (Fig. 3b), minimally processed flows would export ∼5000 t U; even with stricter processing, ∼2000 t U reach export and ∼5000 t U accumulate in *unstable* tailings. **b** In the *intermediate* mobility case, most mines remain at background uranium levels, with a minority operating with modestly elevated (*<*100 ppm) uranium grades. Even under *low* mobility (all mines in the background), ∼2000–3000 t U are exported, with a comparable quantity disposed in unstable tailings. For comparison to all scenarios, we also add a red dotted line that represents the approximately 350 tonnes of uranium that would be present in exports if all shipments from the DRC met the shipping limit of 75 ppm. Colors and symbols after (**a**). Figure produced with Matplotlib^85^.

From modeled uranium grades, reported cobalt grades, and annual cobalt exports, we apply Eq. (1) to estimate total uranium extracted over 2000–2024. We then partition this total between exports and tailings using the pathway assumptions above (Fig. 6). Final results combine three low, intermediate, and high uranium-mobility settings (Fig. 6b), with three hydrometallurgical regimes: (a) a single FAM step only at pH 4, (b) two FAM steps at pH 4 and pH 5.5, (c) two FAM steps and feedstock mineralogy considerations.

In sulfide ores, uranium grades are similar to or higher than those in oxides^53,60^ but reside in distinct minerals under reducing conditions (e.g., uraninite)^51,53^. Accordingly, for sulfide-only operations, we assume uranium is discarded to unstable tailings during upgrade and pyrometallurgy, and for mixed oxide–sulfide operations, we assume 50% of the uranium goes to tailings. This approach likely overstates sulfide impacts, since sulfides are not expected to comprise half of mixed feeds^20^, and some mines may still dissolve mixed ores and process them as oxides^36^. In this mineralogy-affected scenario, where we consider the potential impact of sulfides, we also allow for the oxide fraction of ores at the mine with reported endogenous phosphate removal^20^ to deport all uranium to stable tailings. Cobalt grade, export total, and mineralogical considerations data that combine for our final estimates can be found in our Supplemenary Data 1.

In the low-mobility case, where all mines sit at Copperbelt background (4–7 ppm U), we obtain the minimal quantities at stake. Even paired with maximum plausible in-country removal, we find that *>*1000 t U likely were exported from the DRC. With more realistic processing considerations, total exports range from 1500–2500 t U for the minimal estimate (Fig. 6b). In a scenario where more uranium is removed from exports through incidental means, we find that 3000–4000 tonnes may be left in unstable tailings^42,61^.

The intermediate-mobility scenario is most plausible: most operations are running at background uranium levels, with several mines operating with modestly elevated uranium levels (30–100 ppm U; Fig. 4; Fig. 6b). This small increase nearly doubles the uranium at stake and raises potential exports to over 5000 t U.

Although less likely, the high-mobility case illustrates the risks of overlooking uranium coproduction amid surging cobalt mining in uranium-rich geological systems^62,63^. Most mines would remain under 200 ppm U, unconventional by uranium-industry standards^63,64^, yet the scale of cobalt output implies the DRC would incidentally rank among leading uranium producers. For 2023, modeled exports approach 3500 t U, placing the country within the global top six^4^. This secondhand uranium production mirrors the byproduct nature by which cobalt is recovered from some copper operations: even ores with 50–200 ppm U, when co-mineralized and processed alongside cobalt, can yield consequential uranium production.

### Artisanal mining and uranium estimates

The rise of DRC cobalt production during our study period has received some attention due to the prevalence of artisanal mining. This practice, where local workers, sometimes young children, extract and wash heterogenite by hand in dangerous and unhealthy conditions^65^, all for sub-poverty wages, is broadly considered a humanitarian crisis^56^. The contribution of this sector to the overall DRC cobalt industry is uncertain but likely has fluctuated from representing a majority of production when industrial mining infrastructure was under development prior to 2008^31^, to near or below a 40% share in the years following^25^.

Regardless of its contribution to DRC production, we do not expect underconstrained artisanal production to impact our estimates, as artisanal ores are mined from the same deposits as nearby industrial mines. They are reported at higher cobalt and uranium grades than industrial ores simply because some of the gangue minerals have been removed^13,65^. Artisanal production then enters the industrial supply reported by DRC facilities and is included in the crude cobalt hydroxide or concentrate totals that serve as the basis of our study^25^. Artisanal cobalt that is reported to be leaving the DRC outside of official exports^66^ would only add uranium to the totals we estimate to be extracted and exported outside of nuclear accountancy. The prevalence of artisanal mining, with respect to this study, points to added environmental health and safety concerns of the uranium association with cobalt, as it increases the direct, unprotected contact that local workers have with uranium-enriched materials.

## Discussion

Our results show that in the Democratic Republic of Congo (DRC), the world’s leading source of cobalt, large quantities of uranium are extracted, concentrated, and likely exported in cobalt products. In the Copperbelt, uranium co-occurs with oxidized cobalt ores, and the two behave similarly during hydrometallurgical processing. Absent targeted removal, uranium partitions into cobalt hydroxide. Using a first-order carryover model, combined with geological mapping, mineralization data, and mine-level trade records from 2000–2024, we estimate that approximately 2000–5000 t of natural uranium were exported embedded in cobalt-hydroxide shipments, about 65% of which were sent to Chinese-owned companies that dominate the global cobalt refining market^16^. We further estimate an additional 1000–4000 t accumulated in unstable tailings across the Copperbelt. These estimated ranges arise from the range of processing possibilities combined with the intermediate uranium mobility scenario.

These quantities have implications for nuclear safeguards. In safeguards practice, uranium in ore lies outside accountancy until it is recovered as source material for fissile material production. Thus, uranium carried in cobalt hydroxide can leave the DRC outside declared uranium flows and only enter accountancy when recovered downstream, consistent with the Finnish Kokkola case (2010–2017), where uranium from DRC-origin cobalt products was declared and sold after recovery^22^.

To our knowledge, no other uranium originating from the DRC has been declared to the IAEA. In China’s case, our estimates indicate that the uranium embedded in DRC cobalt-hydroxide imports could have triggered the Additional Protocol requirement to declare annual imports exceeding 10 t from a single non-nuclear-weapon state in every year since 2010 (and by more than ∼50 times in 2024)^67^. Even if all cobalt shipments were meeting the export thresholds of 75 ppm U, the amount of uranium reaching China (~40 tonnes in 2024) would exceed annual reporting limits starting in 2016, illustrating the challenge that the surging cobalt industry presents to nuclear accountancy and safeguards. The Chinese share of the ∼2000–5000 t of undeclared uranium we estimate over 2000–2024 could materially affect national uranium balances and would not be subject to designations of end use, civilian or military^63,68–70^.

Beyond safeguards, health and environmental implications are immediate. Biomonitoring in the Copperbelt has shown elevated uranium in residents and artisanal miners via industrial emissions and direct contact^13,71^. While metal mining broadly raises toxic-metal exposures^72–74^, communities near DRC cobalt operations face uranium-specific hazards, notably radon in poorly ventilated mines and homes^75–77^. Recognizing uranium’s pervasive presence in the cobalt supply chain, it is therefore essential to implement targeted uranium-removal steps across the sector (e.g., post-FAM phosphate precipitation or ion exchange), require transparent reporting with independent assays of cobalt-hydroxide shipments, strengthen tailings controls, and enforce worker protections.

## Methods

### Chemical model of uranium removal during pH manipulation

*Uranium precipitation as hydroxide* – Refinery bleed streams are compositionally complex and vary by ore^26^. We use an equilibrium framework to approximate the onset of uranium precipitation (as uranyl hydroxide) as a function of pH, evaluated at fixed Eh (target ≈ 0.6 V) and under sulfate-dominated complexation consistent with sulfuric-acid leach^21,33^. This plant-agnostic, first-order model captures the principal control, pH, on incidental uranium removal during one- or two-step FAM precipitation, while avoiding uncertain site-specific kinetics and minor-complexation effects.

Because sulfuric acid is used in leach, sulfate dominates aqueous complexation and uranyl occurs primarily as ${{{\rm{UO}}}}_{2}({{{\rm{SO}}}}_{4})_{2}^{2-}$^21,78–80^. We therefore use the U–S–H_2_O equilibrium relations of ref. ^38^. The equilibrium pH is 4.8 for uranium precipitation as uranyl hydroxide (UO_2_(OH)_2_·H_2_O) when [U] = 10^−2^ M and [S] = 10^−1^ M. Using the equilibrium equation, we get an expression for the equilibrium constant, K_eq_:

2 $${{{\rm{K}}}}_{{{\rm{eq}}}}=\frac{{[{{\rm{S}}}{{{\rm{O}}}}_{4}^{2-}]}^{2}}{{{{\rm{UO}}}}_{2}({{{\rm{SO}}}}_{4})_{2}^{2-}}*\, {10}^{3({{{\rm{pH}}}}_{{{\rm{eq}}}}-14)}\approx 3.98\times {10}^{27}.$$

In sulfate-rich bleed streams, > 90% of total U is present as ${{{\rm{UO}}}}_{2}({{\rm{S}}}{{{\rm{O}}}}_{4})_{2}^{2-}$^21,78–80^. Here, we assume $[{{\rm{U}}}{{{\rm{O}}}}_{2}({{\rm{S}}}{{{\rm{O}}}}_{4})_{2}^{2-}]\approx {[{{\rm{U}}}]}_{{{\rm{tot}}}}$. Solving for pH_*eq*_ under typical cobalt-stream conditions^21^:

3 $${{{\rm{pH}}}}_{{{\rm{eq}}}}=14+\frac{1}{3}{\log }_{10}\left(\frac{{[{{\rm{S}}}{{{\rm{O}}}}_{4}^{2-}]}^{2}}{[{{{\rm{UO}}}}_{2}({{{\rm{SO}}}}_{4})_{2}^{2-}]{{{\rm{K}}}}_{{{\rm{eq}}}}}\right)\approx 6.1.$$

To map equilibrium into a smooth removal curve, we approximate the titration behavior with a logistic (sigmoid) function for the dissolved fraction,

4 $${{{\rm{U}}}}_{{{\rm{dissolved}}}}({{\rm{pH}}})=\frac{1}{1+1{0}^{{{\rm{\alpha }}}\left(\right.{{\rm{pH}}}-{{\rm{p}}}{{{\rm{H}}}}_{{{\rm{eq}}}}}},$$

with slope parameter *α* = 2 to reproduce a sharp rise in precipitation near pH_eq_ while keeping negligible removal at the lower pH values typical of single-step FAM.

*Uranium adsorption*—Uranium can also leave the cobalt stream by adsorbing as uranyl (UO^2+^_2_) onto metal oxides and hydroxides that precipitate from other impurities during pH adjustment. Because the precipitation threshold for trace uranium occurs at substantially higher pH than for the bulk impurities^38,39^, and higher than typical FAM setpoints^33,35,55^, adsorption is expected to be the dominant pathway for incidental U removal at low pH.

As with precipitation, the mechanics of uranium adsorption depend on the abundance and surface area of co-precipitated metal (oxy)hydroxides (set by ore impurity levels) and on dissolved co-ions that can enhance or inhibit uranyl binding. Here, we adopt a plant-agnostic, mean-state representation intended to be broadly generalizable to Copperbelt cobalt streams.

Iron is expected to be the dominant scavenger of uranium because it is a bulk contaminant in Copperbelt ores^36^ and precipitates at relatively low pH^39^, generating substantial iron (oxy)hydroxides during FAM removal. We parameterize uranyl adsorption to ferric (oxy)hydroxides using the combined modeling–experimental dataset of ref. ^40^, adopting their goethite (FeO(OH)) curve. Although amorphous Fe(OH)_3_ may commonly form during FAM, the experiments of 40 were conducted in simplified solutions with few competing ions; the goethite curve exhibits a more gradual, pH-dependent uptake and thus provides a conservative, plant-agnostic approximation for refinery conditions. This adsorption function serves as our baseline model.

Naturally occurring phosphate minerals can release phosphate ions to refinery streams^35,55^, and phosphate is known to enhance uranyl adsorption onto metal (oxy)hydroxides^41^. Although we found no studies quantifying phosphate-enhanced uranyl uptake on iron (oxy)hydroxides, ref. ^41^ report a threshold-type co-sorption on Al_2_O_3_. Once a critical phosphate concentration is reached, uranyl uptake increases at an approximately constant rate. Guided by this behavior, we implement a phosphate-enhanced variant of our adsorption model in which, above an assumed threshold, uranyl uptake is increased by up to 50% relative to the baseline goethite curve (capped at full uptake). This conservative cap reflects competition in process solutions and the likelihood that phosphate partly precipitates metal phosphates^35^, reducing the free phosphate available to promote adsorption.

*Composite model* – Adsorption dominates uranium removal at the low pH used for FAM. Because cobalt refining begins with acidic leach (pH ≈ 1) and then incrementally raises pH to strip impurities^20,33,35^, often in streams rich in iron^36^, we expect adsorption to prevail over most of the operating window. Accordingly, our composite curve first applies the adsorption model (baseline or phosphate-enhanced) to compute the fraction of uranium removed and then applies the precipitation model to the residual dissolved uranium to capture additional removal at higher pH (Fig. 2b). We hold K_*eq*_ fixed and do not re-solve speciation after adsorption lowers the uranium concentration; this assumption likely overestimates precipitation (and thus total incidental removal), leading to a conservative underestimate of uranium in exports, especially at higher pH scenarios.

### Imports analysis for targeted uranium removal

Refineries can deliberately remove dissolved uranium by either (i) dosing phosphoric acid after FAM to precipitate uranium as insoluble phosphates or (ii) installing strong-base ion-exchange (IX) systems that capture uranyl–sulfate complexes^21^. Both technologies require the import of specific compounds. We therefore use customs data on IX resins and phosphoric acid to infer adoption at the mine level.

For phosphoric acid (HS 28092000), the dataset contains *>*2000 entries by mining and refining firms. Because phosphoric acid has multiple process roles (e.g., adjusting leach selectivity^61^), we use imported volumes normalized by cobalt output as a heuristic to identify deliberate uranium removal.

Following reporting that elevated radioactivity led Glencore’s Kamoto Copper Company (KCC) to suspend operations in 2019 and implement uranium-removal measures^81^, we observe a step-change in KCC phosphoric-acid purchases beginning in 2019: at least ∼1,000,000 L per ∼25,000 t Co exported (i.e., ∼40 L per t Co). We adopt this as a first-order benchmark (Fig. 3a), recognizing that reagent demand varies with ore chemistry. Applying this threshold across mines, only two refineries consistently meet or exceed this total over the study period; one mine begins purchases in 2023, and three additional mines begin in 2024.

For ion-exchange (IX) systems, adoption should be visible in customs data as large purchases of strong-base anionic resins targeted at uranium removal. While small IX resin purchases may be common to refill existing systems in mining infrastructure, we are looking for newly-established resin beds targeted at removing uranium. Estimates place the initial investment for this first fill of IX resins at 1,712,000 USD^21^, which we adopt as a threshold to identify candidates for uranium removal. Using Export Genius data for ion-exchange resins (HS 39140000) imported into the DRC since 2019, we identify *>*150 entries. Only one purchase approaches the expected IX capital magnitude (Fig. 3b). That facility processes copper, not cobalt^82^, so this acquisition would not remove uranium from cobalt streams. The remaining mining-related resin imports are $10,000–$100,000 lots, consistent with effluent-treatment uses rather than uranium-specific IX^83^.

### Model of uranium mobility in cobalt-bearing deposits

We simulate preferential uranium mobility along faults using a process-agnostic, two-dimensional convolution model on gridded uranium grades. This choice avoids committing to diffusion- or advection-dominated transport, which are not constrained at the regional scale. Inputs comprise (i) a binary raster of fault presence/absence from ref. ^29^ and (ii) a uranium-grade raster. The uranium raster is initialized to the Copperbelt background everywhere, with pixels coincident with mapped uranium deposits^8^ set to 1000 ppm. Both rasters use a 100 m pixel size (adjustable).

We first dilate the fault mask by one iteration with a square structuring element of connectivity one. Mobility is then simulated iteratively. At each iteration, we convolve the uranium raster with a Gaussian kernel and set each pixel to *µ *+ *σ* of the kernel window to emphasize spread in areas of high local heterogeneity. We cap the blurred field at the iteration’s input maximum so that grades never exceed the 1000 ppm source value.

To accentuate transport along faults, we multiply the blurred field by a fault conductivity raster: fault pixels take the value of 1, non-fault pixels take the inverse of a contrast factor. We use a contrast of 1.2, which confines high grades predominantly to the fault network (SI Fig. 1). After this weighting, we reset deposit pixels to 1000 ppm and floor all pixels at the background grade before proceeding to the next iteration. Results shown use 10 iterations for the intermediate-mobility case and 50 iterations for the high-mobility case. To assign a uranium grade to each operation, we compute the mean modeled grade within the mining concession, masked to formations considered cobalt-bearing.

### Reporting summary

Further information on research design is available in the Nature Portfolio Reporting Summary linked to this article.

## Supplementary information

Supplementary Information

Description of Additional Supplementary Files

Supplementary Data 1

Supplementary Data 2

Supplementary Data 3

Supplementary Data 4

Reporting Summary

Transparent Peer Review file

## Data Availability

The authors declare that the data relating to cobalt mining production in the DRC, as well as imports of phosphoric acid and ion exchange resins supporting the findings of this study, are available within the Supplementary Data. Geochemical ground truths for uranium grade are available at 10.1016/j.mineng.2024.108993. African country boundaries for maps are available at https://hub.arcgis.com/datasets/geoduck::africa-boundaries/about. Cobalt permissive tracts in the DRC are available at https://pubs.usgs.gov/sir/2010/5090/t/downloads/sir2010-5090T_GISdata.zip. Spatial files for Katangan geological units and faults are available at https://geocatalogue.africamuseum.be/geonetwork/srv/api/records/BE-RMCA-EARTHS-023779. Spatial files for mining permit boundaries are available at https://data.globalforestwatch.org/datasets/84fbbcc10c9f47f890750dd42426cbd2_18/explore?location=-9.398000%2C25.386602%2C8. Southern DRC uranium deposit locations are available at 10.2113/gsecongeo.76.1.56. Annual global cobalt production data are available at https://www.usgs.gov/centers/national-minerals-information-center/cobalt-statistics-and-information

## Code availability

The code developed for this article is available at https://github.com/rmanzuk/DRC_U_estimate.
