# Supplementary material for: Uranium in cobalt-hydroxide exports from the Democratic Republic of the Congo
Source: Nat Commun. 2026 Jul 30;17:7415. doi: 10.1038/s41467-026-75910-z (PMC13424566; doi:10.1038/s41467-026-75910-z)
Supplement: Supplementary file 1 — Supplementary Information [file 41467_2026_75910_MOESM1_ESM.pdf]

Supplementary Information: Uranium in cobalt-hydroxide exports from the Democratic Republic of the Congo

Ryan A. Manzuk[1,2,*], Sébastien Philippe[2]

[1] Program on Science and Global Security, Princeton University, Princeton, NJ, 08542, USA
[2] Department of Nuclear Engineering and Engineering Physics, University of Wisconsin-Madison, Madison, WI, 53711, USA

[*]correspondance to rmanzuk@princeton.edu

This supplementary information file accompanies the manuscript *Uranium in cobalt-hydroxide exports from the Democratic Republic of the Congo* published in Nature Communications. In the study, we use a simple model of uranium mobility preferentially along faults to constrain uranium prevalence in commercial cobalt mines. In this Supplementary Information file, we give one additional figure and caption to display modeling results and parameters.

Uranium mobility model sensitivity

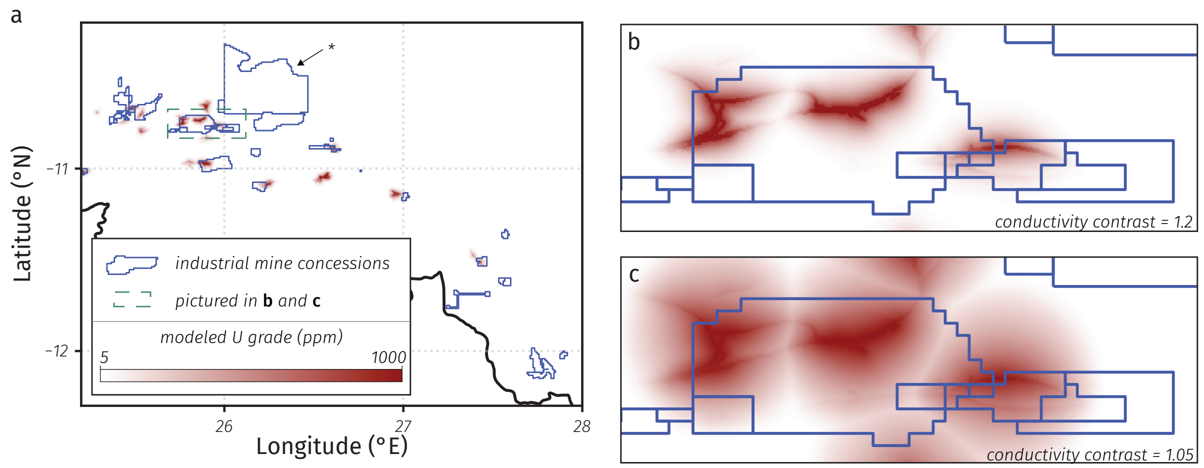

Supplementary Information Fig. 1: Uranium-mobility model results and parameters. (**a**) Because cobalt- and uranium-bearing deposits preferentially align with faults, the model produces elevated uranium zones that often overlap industrial concessions in this high-mobility scenario. By contrast, the mine used to calculate the uranium baseline grade in the manuscript, indicated by arrow and star, is one of the furthest industrial operations from a concentrated uranium deposit or an associated fault conduit. We thus use ore uranium content data from this locality as a constraint on background grade in the region (Fig. 4). Other industrial mining concessions are expected to have elevated grades due to local uranium deposits and faults. Administrative boundaries from the Global Administrative Areas database (GADM; https://gadm.org) (**b,c**) Sensitivity to the fault–matrix conductivity contrast: a contrast of 1.2 confines high grades primarily to the fault network (used in main results), whereas a weaker contrast (1.05) yields unrealistically widespread and symmetrical high grades. Figure produced with Matplotlib[1], GeoPandas[2], and Rasterio (https://rasterio.readthedocs.io).

References

[1] J. D. Hunter. Matplotlib: A 2d graphics environment. *Computing in Science & Engineering*, 9(3):90–95, 2007. doi: 10.1109/MCSE.2007.55.

[2] Kelsey Jordahl, Joris Van den Bossche, Martin Fleischmann, Jacob Wasserman, James McBride, Jeffrey Gerard, Jeff Tratner, Matthew Perry, Adrian Garcia Badaracco, Carson Farmer, Geir Arne Hjelle, Alan D. Snow, Micah Cochran, Sean Gillies, Lucas Culbertson, Matt Bartos, Nick Eubank, maxalbert, Aleksey Bilogur, Sergio Rey, Christopher Ren, Dani Arribas-Bel, Leah Wasser, Levi John Wolf, Martin Journois, Joshua Wilson, Adam Greenhall, Chris Holdgraf, Filipe, and François Leblanc. geopandas/geopandas: v0.8.1, July 2020. URL https://doi.org/10.5281/zenodo.3946761.
