# Supplementary material for: Uranium in cobalt-hydroxide exports from the Democratic Republic of the Congo
Source: Nat Commun. 2026 Jul 30;17:7415. doi: 10.1038/s41467-026-75910-z (PMC13424566; doi:10.1038/s41467-026-75910-z)
Supplement: Supplementary file 2 — Description of Additional Supplementary Files [file 41467_2026_75910_MOESM2_ESM.pdf]

Description of Additional Supplementary Files

File Name: Supplementary Data 1
Description: Project-by-project annual cobalt export volumes from DRC operations, with operator and ownership metadata, ore type, and feedstock characterization. Used to attribute downstream feedstock flows to specific mining operations.

File Name: Supplementary Data 2
Description: Annual DRC cobalt exports aggregated by feedstock category, in tonnes Co contained. Provides a higher-level summary view of the project-level data in File S2.

File Name: Supplementary Data 3
Description: Transaction-level DRC import records for ion-exchange resins (HS 39140000), used as a proxy for hydrometallurgical reagent flows into cobalt processing facilities. Quantity fields are partially populated (many declarations report value only).

File Name: Supplementary Data 4
Description: Transaction-level DRC import records for phosphoric and polyphosphoric acids (HS 28092000), enriched with attribution to specific cobalt mining or processing operations where the importer or delivery address could be matched. The 'Mine' field is blank for non-mining importers (e.g., breweries, beverage manufacturers).
