# Supplementary material for: Uranium in cobalt-hydroxide exports from the Democratic Republic of the Congo
Source: Nat Commun. 2026 Jul 30;17:7415. doi: 10.1038/s41467-026-75910-z (PMC13424566; doi:10.1038/s41467-026-75910-z)
Supplement: Supplementary file 8 — Transparent Peer Review file [file 41467_2026_75910_MOESM8_ESM.pdf]

nature portfolio

Peer Review File

Uranium in cobalt-hydroxide exports from the Democratic Republic of the Congo

Corresponding Author: Dr Ryan Manzuk

This file contains all reviewer reports in order by version, followed by all author rebuttals in order by version.

Version 0:

Reviewer comments:

Reviewer #1

(Remarks to the Author)
Reviewer Comments: Nature Communications
Uranium in cobalt hydroxide exports from the Democratic Republic of the Congo
R.A. Manzuk, S. Phillippe

This is a novel paper that addresses a significant safety, toxicity, and nuclear accounting issue relating to the deportment of uranium in cobalt hydroxide product exported from the DRC. Few direct measurements of uranium content are available, so this study has used geochemical modelling in combination with export data to estimate the quantities of uranium exported from the country as well as that discarded in tailings repositories within the country.

The paper is very well written and the study is comprehensive and appears generally sound, although some of the estimates appear high. The topic is important and, to date, has not been widely addressed outside of Southern Africa.

Key results
• For the period 2020–2024, some 2–5 kt U were exported from DRC in cobalt hydroxide product, of which a significant portion was to China.
• Less than 10% of this material was declared and accounted for under international standards.
• Up to 3 kt U was discarded in unstable tailings within the country during this period.
• In addition to serious human and environmental safety and toxicity issues, this lack of nuclear accounting presents potential imbalances in international holdings.

Validity
The expertise of this reviewer lies in the processing technologies discussed and removal mechanisms assessed for uranium. This reviewer does not have adequate expertise to critically assess the modelling approach used. Nevertheless, the results are generally compatible with general metallurgical and geochemical knowledge regarding uranium deportment in this region. Some of the estimates of U exports appear high – see later comments: this may be owing to the “smoothing effect” inherent in the modelling approach. The model considers only cobalt mineralogy and does not take into account copper mineralogy (such as pseudomalachite).

Significance
Owing to political and transparency issues within DRC with respect to uranium, and its close relationships with several “rogue states”, there has been little spotlight on the human and environmental damage caused by radiation associated with the presence and illegal mining of uranium in the country. In recent years, shipments of cobalt hydroxide, which contain U that has deported with cobalt from the ores, have resulted in transport of radioactive material around the globe, mainly to China. This uranium is largely unreported, which means that holdings of uranium by recipient countries may be uncertain. This article has made a good effort to quantify and define the deportment of uranium from DRC. These data can be used in support of capital projects to address uranium removal from the cobalt hydroxide product and retain the uranium in in-country tailings storage facilities in highly stable form.

Data and methodology
The validity of the modelling approach is not assessed in this review, owing to lack of expertise of the reviewer in the detail

methodology.

All other data and methods are generally sound, although some caution is required in data interpretation owing to the effect of differing copper mineralogies in the region – see below comments.

Suggested improvements
An annotated copy of the manuscript is provided, with suggested corrections, as summarised below.
• The abstract should contain no references – see Guidelines for Authors.
• Line 150: It should be noted that another U removal route occurs in the main copper leaching step. In several circuits, where Cu is present as pseudomalachite, U is precipitated as very stable autunite and deports to the leach residue. For example, at Mutanda Mining, no phosphoric acid is added, but the U content of the cobalt product is extremely low owing to this mechanism. The text states that endogenous phosphate is unlikely to achieve substantial uranium removal: this is not true and is site-specific, dependent on the copper mineralogy.
• With respect to using TFM as the baseline, it is stated that this mine is far away from the main uranium locations. However, it is a lot closer to Shinkolobwe than many of the other mines (such as Etoile, cited as using phosphoric acid). It would be useful to annotate these locations on one of the maps.
• With respect to characterisation of the in-country precipitation residues, it is stated that these are mainly unstable. In fact, when using phosphoric acid, the main precipitate is understood to be autunite, in which U is in a more stable form than in the original mineral.
• Regarding a second precipitation step at higher pH, this is typically carried out to recover residual copper from the SX raffinate that is treated through the FAM circuit. This second-stage precipitate would be recycled to the main copper circuit for copper recovery, so U would be releached in this step. Use of a higher pH for precipitation therefore does not really present a viable option for U removal.
• Line 282: Units of concentration should be provided.
• Line 321: Define Keq.
• Lines 324–340: Suggest including full company names for clarity. The shortened/common names are used here.
• Lines 341–348: While imports of phosphoric acid can be stoichiometrically correlated with Co(OH)2 production, the same is not true for the use of IX resins for U removal. Owing to the nature of the technology, there is a once-off first-fill order of resin on commissioning. This can be used for > 20 years. In subsequent years, only small "top-up" resin purchases may be made. A brief discussion/clarification of how the IX resin requirements were estimated could be helpful.
Note for interest: One commercial IX system was installed at Metalkol in DRC for U removal in about 2018. However, it took so long to commission that the (incorrectly specified) chloride form of the resin corroded the inside of the columns before it was even started up, and eventually phosphoric acid was used instead, as this proved a less-expensive option. I am not aware of any formal reference to this.
• Since ~ 2018, the DRC legislated limit is 75 ppm U in cobalt hydroxide exports. This should be stated. [Whether this target is actually adhered to or monitored (or can even be accurately measured) is another matter ☹.] However, for the 2024 data, using 160 kt Co exported as hydroxide and assuming 30% Co content of the hydroxide product, 75 ppm U translates to 40 t U. The estimates of 2000–5000 t U over the 2020–2024 period therefore seem high.
• Guidelines specify a maximum of 70 references. Please confirm with editor if this larger number of references is acceptable.

Clarity and context
The paper is exceptionally well written and generally very clear.

To conform to conventional practice in science/engineering papers, the following are suggested:
• SI number format should be used throughout the text, except for financial figures. A space is used for the thousand separator for numbers > 9999.
• Abbreviations should only be defined if they will be used at least three times in the text. Once an abbreviation is defined, the abbreviation should be used throughout.
• It is suggested to use consistent units of kt (or t) in all relevant graphs to make comparison easier.
• Chemical formulae (Methods section) should not be italicised.

References
To the best of this reviewer's knowledge, all relevant prior studies are adequately and appropriately referenced.

Reviewer #2

(Remarks to the Author)
This manuscript addresses an ambitious, timely, and highly complex issue: the fate of uranium associated with cobalt ores from the Copperbelt of the Democratic Republic of Congo (DRC), and its de facto export through official cobalt supply chains. By bridging economic geology, extractive metallurgy, international trade, nuclear safeguards, and environmental risk, the study tackles a genuine blind spot in current international accounting and regulatory frameworks.
The manuscript presents several noteworthy and original results. Most prominently, the authors quantify, for the first time at this scale, the magnitude of uranium exports embedded in cobalt hydroxide products over a multi-decadal period (2000–2024), combining geological, mineralogical, geochemical, metallurgical, and trade datasets. The integration of mine-level export records with geological mapping and process-based modelling is particularly compelling and represents a major strength of the study. This holistic approach allows the authors to move beyond speculative assessments and provide first-order quantitative constraints on uranium flows that have remained largely unaccounted for.
The work is clearly of high significance to the field and to several related disciplines. For geoscientists and economic

geologists, it provides new insights into uranium–cobalt co-mineralization and the downstream behaviour of uranium during hydrometallurgical processing. For the nuclear safeguards and policy communities, it raises important questions regarding undeclared uranium movements within legal supply chains. Compared to the established literature, the manuscript goes well beyond previous descriptive or localized studies by integrating heterogeneous datasets across spatial, temporal, and disciplinary scales. While individual components of the study build on well-established concepts, their synthesis into a coherent, system-level analysis is both original and impactful.
Overall, the data presented convincingly support the main conclusions and claims. The analytical framework is logically constructed, and the progression from geological observations to trade-flow estimates is clear and transparent. The conclusions regarding the scale of uranium export, the limited declaration under international safeguards, and the lack of systematic uranium removal practices are well grounded in the presented evidence. Some interpretations would benefit from additional contextualization—particularly for non-specialist readers—but these points concern clarity and framing rather than the validity of the results.
The data analysis and interpretation are sound. The methods applied to integrate geological, mineralogical, and trade datasets are appropriate and consistent with current standards in the field. No major flaws were identified that would prohibit publication. Any issues raised relate primarily to the need for clearer contextual background, rather than to weaknesses in the analysis itself, and can be addressed through moderate revisions.
The methodology is robust and well suited to the objectives of the study. The authors demonstrate a strong command of both the geological system and the industrial processing chain, and the modelling approach used to estimate uranium carryover is reasonable and clearly justified. The Methods section is generally detailed enough to ensure reproducibility, with clear descriptions of data sources, assumptions, and analytical steps. Minor additions or clarifications in specific places would further enhance transparency but do not require substantive methodological changes.
Given the intrinsic complexity of the system investigated, my main recommendation is to further strengthen and expand the Introduction using well-established and readily accessible contextual information. In particular, a short discussion of the evolution and relative importance of artisanal and informal mining in the DRC Copperbelt over the 2000–2024 period would help contextualize uncertainties in production statistics and material traceability. Likewise, a concise synthesis of existing IAEA findings and concerns regarding uranium in the DRC—especially in relation to cobalt-associated flows—would more explicitly anchor the manuscript within the existing safeguards and regulatory discourse. Brief introductory context on cobalt processing infrastructure in Katanga would also help readers understand where uranium partitioning into cobalt hydroxide is most likely to occur along the value chain.
Finally, translating the quoted uranium quantities into energy-equivalent terms would significantly enhance the broader societal relevance of the results. A short discussion on uranium speciation in heterogenite, particularly at high uranium concentrations, would further strengthen the geochemical interpretation and complement the existing discussion focused on Fe oxyhydroxides.

Detailed comments
I strongly encourage the authors to consider the following additions to the Introduction:
1. Artisanal and informal mining in the DRC (2000–2024)
Since the analysis explicitly covers the period 2000–2024, it would be very useful to include a short discussion of the artisanal and informal mining sector in the DRC Copperbelt. In particular:
o How has this sector evolved over the considered period?
o What proportion of total Copperbelt cobalt production does it represent, at least in broad terms?
This information would help contextualize uncertainties related to production statistics, traceability, and undeclared material flows.
2. IAEA findings on uranium in the DRC and cobalt-associated flows
A short synthesis of the main conclusions and concerns raised by the IAEA regarding uranium in the DRC would be highly valuable. In particular:
o What is currently known and officially reported about uranium trafficking or undeclared production?
o What has been reported specifically in relation to cobalt-associated uranium during the 2000–2024 period?
This would anchor the manuscript more explicitly within the existing safeguards and regulatory discourse.
3. Cobalt processing infrastructure in Katanga
The manuscript would benefit from 2–3 introductory lines describing the processing units and refineries that are actually capable of treating cobalt ores in the Katanga region. This would help readers better understand where and how uranium partitioning into cobalt hydroxide is likely to occur along the value chain.
4. Energy-equivalent context for uranium quantities
Given the quantities quoted in lines 16–17, it would be extremely useful to briefly translate these figures into energy-equivalent terms, i.e.:
o What do these uranium masses correspond to in terms of potential electricity generation or nuclear fuel use?
This would greatly enhance the broader societal relevance and impact of the results.
5. Uranium speciation in heterogenite
Finally, I recommend adding a short discussion on the speciation of uranium in heterogenite, particularly at high U concentrations. Specifically:
o Is uranium considered to be primarily adsorbed, and if so, in the form of which complexes?
o Is co-precipitation invoked for the highest U contents?
Adsorption is discussed on page 11, but the focus there is mainly on Fe oxy-hydroxides; extending this discussion to heterogenite would be highly valuable.

Minor and Specific Comments
• Lines 9–10: Please indicate what fraction of global reserves this corresponds to.

• Line 15: It would also be helpful to specify the percentage here.
• Line 42: “an oxyhydroxyde” → an oxyhydroxide.
• Line 43: Please specify the quantity more precisely.
• Line 74: One reason for highly U-enriched heterogenite is that, in the primary (sulfide) mineralization, uraninite is frequently associated with Co–Ni–Cu sulfides in deposits known for overall U enrichment. This point could be explicitly stated.
• Line 102: Suggested rephrasing: “They occur in the same mineral only in the oxidized ore.”

Reviewer #3

(Remarks to the Author)
This is an interesting and sound paper, which focuses on a little-studied theme, relevant for both pollution, public health and nuclear safeguards.

I don't have a lot of comments, safe for the geologic presence of uranium in the Copperbelt. I fully agree that fault zones may contribute, since the mineralized rocks are exposed, to the migration of uranium. But the role of fault zones is more complex than sketched in the paper. U primary stock in the sedimentary basin is geologically “old” (in the order of 800 Myr, roughly contemporaneous with the Cu-Co deposit, Decrée et al., 2014) and fault zones contributed to multiphase migration of this metal along the 800 Myr-long geological history, and notably during the Lufilian orogeny (Decrée et al., 2011). Intrinsic U mobility in oxidizing conditions, prevailing since about 80 Myr in the study area (De Putter and Ruffet, 2020), has allowed its migration and coprecipitation with oxidized cobalt ore (heterogenite) – a process likely enhanced in the last 10 Myr, as rightly stated in the paper. This is just to emphasize that there are Neoproterozoic fault-related subvertical “primary” U deposits (such as Shinkolobwe), comprising a surprisingly complex metal paragenesis: Au + U + Se + Mo + Ni + PGE, and more. Whether such a deposit is at the origin of all the other showings in Katanga, through migration and dissemination of this “primary” U stock is still open for debate. This geological nitpicking doesn’t affect the assumption that recent exposure of the deposits to oxidizing conditions have fostered U lateral migration, either from Shinkolobwe-type deposit(s) or from other “secondary” reconcentrated stocks of U along the last 800 Myr.

In this context, I would turn the sentence “cobalt ores co-occur with uranium” (line 244) the other way: “uranium co-occurs with oxidized cobalt ore”.

The sentence “DRC copper deposits commonly contain economic grades of cobalt” (line 37) makes me feel uncomfortable. The Copperbelt hosts a unique type of copper and cobalt deposit, the only one on Earth where cobalt is enriched to the point that it is a commodity exploited on its own. In other deposits, it is generally a by-product of another metal (typically in ultramafic Ni-Co deposits).

In a broader way, the sentence “cobalt’s rise via the copper boom” (line 239) is not fully correct; there were copper booms without a cobalt rise in the past. The present cobalt rise is mostly due to specific needs in this metal since WWII (De Putter 2019, no need to cite!) even if, in some instances, end-products (as e-powered cars) require both metals.

Line 33 and fig. 1: I don’t like “bands” of Roan sediments (or of metalliferous rocks): “outcrops” would be much better.

Last detail: I didn’t check if Shinko was closed “80 years ago” (line 26) … I would have said later but I may be wrong.

Version 1:

Reviewer comments:

Reviewer #1

(Remarks to the Author)
With respect to the revised manuscript and responses to the issues raised by all three reviewers, I am very happy with the second version and have no hesitation in accepting this revised manuscript for publication. All comments were more than adequately addressed and I believe that these modifications improve an already excellent paper and ensure full clarity where there was some mis-phrasing or incomplete statements.

I congratulate the authors on a very thorough and comprehensive study of a very important topic. I believe that this will become a benchmark paper and be widely cited in the upcoming years.

(Remarks on code availability)
See comments on first review.

Reviewer #2

(Remarks to the Author)
All the questions and minor points of imprecision I previously identified have been carefully addressed, and I sincerely thank the authors for their thorough revisions and for taking my comments into account.

(Remarks on code availability)

Reviewer #3

(Remarks to the Author)
My comments have been taken into consideration and the paper is now improved.

(Remarks on code availability)
My comments have been addressed and improvements made. As far as I've seen, other comments by other reviewers have been also addressed. In my opinion, this improved version might be published as it is. It is certainly a relevant topic right now given the importance of nuclear material supply chains and the risk of conflict escalation, in Iran and the Middle East. Besides its scientific qualities, it appears in a context which adds to its relevance and intrinsic scientific value.

Open Access This Peer Review File is licensed under a Creative Commons Attribution 4.0 International License, which permits use, sharing, adaptation, distribution and reproduction in any medium or format, as long as you give appropriate credit to the original author(s) and the source, provide a link to the Creative Commons license, and indicate if changes were made.
In cases where reviewers are anonymous, credit should be given to 'Anonymous Referee' and the source.
The images or other third party material in this Peer Review File are included in the article's Creative Commons license, unless indicated otherwise in a credit line to the material. If material is not included in the article's Creative Commons license and your intended use is not permitted by statutory regulation or exceeds the permitted use, you will need to obtain permission directly from the copyright holder.
To view a copy of this license, visit https://creativecommons.org/licenses/by/4.0/

REVIEWER COMMENTS TO AUTHORS

Reviewer 1

General comments

This is a novel paper that addresses a significant safety, toxicity, and nuclear accounting issue relating to the deportment of uranium in cobalt hydroxide product exported from the DRC. Few direct measurements of uranium content are available, so this study has used geochemical modelling in combination with export data to estimate the quantities of uranium exported from the country as well as that discarded in tailings repositories within the country.

The paper is very well written and the study is comprehensive and appears generally sound, although some of the estimates appear high. The topic is important and, to date, has not been widely addressed outside of Southern Africa.

Response: We thank the reviewer for their careful reading and positive assessment of the manuscript. We appreciate the recognition of the novelty and importance of the topic. Below, we address each recommended improvement in detail.

Key results
• For the period 2020–2024, some 2–5 kt U were exported from DRC in cobalt hydroxide product, of which a significant portion was to China.
• Less than 10% of this material was declared and accounted for under international standards.
• Up to 3 kt U was discarded in unstable tailings within the country during this period.
• In addition to serious human and environmental safety and toxicity issues, this lack of nuclear accounting presents potential imbalances in international holdings.

Validity
The expertise of this reviewer lies in the processing technologies discussed and removal mechanisms assessed for uranium. This reviewer does not have adequate expertise to critically assess the modelling approach used. Nevertheless, the results are generally compatible with general metallurgical and geochemical knowledge regarding uranium deportment in this region. Some of the estimates of U exports appear high – see later comments: this may be owing to the "smoothing effect" inherent in the modelling approach. The model considers only cobalt mineralogy and does not take into account copper mineralogy (such as pseudomalachite).

Significance
Owing to political and transparency issues within DRC with respect to uranium, and its close relationships with several "rogue states", there has been little spotlight on the human and environmental damage caused by radiation associated with the presence and illegal mining of uranium in the country. In recent years, shipments of cobalt hydroxide, which contain U that has deported with cobalt from the ores, have resulted in transport of radioactive material around the

globe, mainly to China. This uranium is largely unreported, which means that holdings of uranium by recipient countries may be uncertain. This article has made a good effort to quantify and define the deportment of uranium from DRC. These data can be used in support of capital projects to address uranium removal from the cobalt hydroxide product and retain the uranium in in-country tailings storage facilities in highly stable form.

Data and methodology
The validity of the modelling approach is not assessed in this review, owing to lack of expertise of the reviewer in the detail methodology.

All other data and methods are generally sound, although some caution is required in data interpretation owing to the effect of differing copper mineralogies in the region – see below comments

Clarity and context
The paper is exceptionally well written and generally very clear.

References
To the best of this reviewer's knowledge, all relevant prior studies are adequately and appropriately referenced.

Recommended improvements

The abstract should contain no references – see Guidelines for Authors.

Response: We have removed references from the abstract in accordance with the journal guidelines.

Line 150: It should be noted that another U removal route occurs in the main copper leaching step. In several circuits, where Cu is present as pseudomalachite, U is precipitated as very stable autunite and deports to the leach residue. For example, at Mutanda Mining, no phosphoric acid is added, but the U content of the cobalt product is extremely low owing to this mechanism. The text states that endogenous phosphate is unlikely to achieve substantial uranium removal: this is not true and is site-specific, dependent on the copper mineralogy.

Response: We appreciate the reviewer's first-hand expertise regarding uranium removal in copper circuits and agree that this is an important mechanism to acknowledge.

We have revised the text to clarify that endogenous phosphate associated with copper mineralogy (e.g., pseudomalachite) can, under site-specific conditions, precipitate uranium as stable autunite during leaching. We now explicitly note the Mutanda Mining case and cite the relevant reference (Crundwell).

At the same time, the available geological literature does not indicate widespread phosphate-controlled uranium immobilization across the Copperbelt, suggesting that Mutanda likely represents

a site-specific mineralogical context rather than a Copperbelt-wide condition. In addition, iron and aluminum phases may compete for available phosphate prior to autunite precipitation, which could limit this mechanism in many deposits. We also identified a 2021 environmental report from Mutanda (now cited) indicating that phosphoric acid may need to be added to the process line to achieve effective uranium removal, further underscoring that endogenous phosphate alone is not universally sufficient.

To address this variability conservatively and transparently, we have broadened our former "sulfates considered" scenario into a more general "mineralogy considerations" scenario. In this scenario, we assume that all oxide-ore uranium at Mutanda deports to stable tailings. Even under this conservative assumption, our core estimates and overall conclusions remain materially unchanged.

With respect to using TFM as the baseline, it is stated that this mine is far away from the main uranium locations. However, it is a lot closer to Shinkolobwe than many of the other mines (such as Etoile, cited as using phosphoric acid). It would be useful to annotate these locations on one of the maps.

Response: We agree that the Tenke Fungurume Mine is geographically closer to Shinkolobwe than other mines. However, our geological analysis indicates that it is not situated along or near known fault conduits linking it to a concentrated uranium deposit (including Shinko).

To improve clarity, we have added annotations for COMMUS and Etoile to Extended Data Fig. 2a, and updated the caption accordingly. These mines are expected to have elevated uranium grades based on local geology. We also note that these facilities have recently imported significant quantities of phosphoric acid, suggesting new remediatio responses to elevated uranium levels.

With respect to characterisation of the in-country precipitation residues, it is stated that these are mainly unstable. In fact, when using phosphoric acid, the main precipitate is understood to be autunite, in which U is in a more stable form than in the original mineral.

Response: We agree and this has been our understanding all along. In the main text, we now explicitly state that uranium removed via phosphoric acid precipitation is present in a stable autunite mineral form.

In the abstract, we have revised the wording to avoid implying that all uranium remaining in-country is unstable. The revised sentence clarifies that the instability estimate refers to uranium not deliberately stabilized through phosphate precipitation.

Regarding a second precipitation step at higher pH, this is typically carried out to recover residual copper from the SX raffinate that is treated through the FAM circuit. This second-stage precipitate would be recycled to the main copper circuit for copper recovery, so U would be releached in this step. Use of a higher pH for precipitation therefore does not really present a viable option for U removal.

Response: We thank the reviewer for this important clarification. This point further reinforces our central argument that, unless deliberate and targeted removal steps are implemented, uranium will generally follow cobalt through the precipitation pathway.

We have revised the manuscript to explicitly state that uranium captured in a second, higher-pH precipitation stage would typically be recycled to the main copper circuit and re-leached. We also clarify that durable uranium removal via this pathway is operationally unlikely.

Line 282: Units of concentration should be provided.

Response: We now indicate that the concentrations are in molar units.

Line 321: Define Keq.

Response: We defined Keq explicitly as the equilibrium constant.

Lines 324–340: Suggest including full company names for clarity. The shortened/common names are used here.

Response: We have replaced abbreviated company names with full company names throughout the manuscript.

Lines 341–348: While imports of phosphoric acid can be stoichiometrically correlated with Co(OH)2 production, the same is not true for the use of IX resins for U removal. Owing to the nature of the technology, there is a once-off first-fill order of resin on commissioning. This can be used for > 20 years. In subsequent years, only small "top-up" resin purchases may be made. A brief discussion/clarification of how the IX resin requirements were estimated could be helpful.

Response: We are happy to clarify this point as ruling out the implementation of IX systems is an important element of our argument.

The threshold that we adopted of 1,712,000 USD comes from the Dempers reference cited in our manuscript and specifically refers to the first-fill order pointed to by the reviewer. We have clarified the language in the manuscript to acknowledge that small resin orders may be common, but because we are looking for the establishment of new resin beds to counter the uranium problem, we can specifically look for these large orders.

Because we observe phosphoric acid imports during the study period but no large first-fill IX resin purchases, we infer that phosphoric acid has been the primary method adopted by facilities implementing uranium control measures.

We have clarified this threshold in both the main text and Extended Data Fig. 1

Note for interest: One commercial IX system was installed at Metalkol in DRC for U removal in about 2018. However, it took so long to commission that the (incorrectly specified) chloride form of the resin corroded the inside of the columns before it was even started up, and eventually phosphoric acid was used instead, as this proved a less-expensive option. I am not aware of any formal reference to this.

Response: We thank the reviewer for this insight. While we are unable to identify a formal reference, this account is consistent with our conclusion that phosphoric acid precipitation has emerged as the preferred uranium control method in practice.

Since ~ 2018, the DRC legislated limit is 75 ppm U in cobalt hydroxide exports. This should be stated. [Whether this target is actually adhered to or monitored (or can even be accurately measured) is another matter.] However, for the 2024 data, using 160 kt Co exported as hydroxide and assuming 30% Co content of the hydroxide product, 75 ppm U translates to 40 t U. The estimates of 2000–5000 t U over the 2020–2024 period therefore seem high.

Response: We agree that the regulatory limit of 75 ppm should be acknowledged, and we have added a sentence in the manuscript describing this threshold in relation to our findings. As the reviewer correctly notes ("Whether this target is actually adhered to or monitored (or can even be accurately measured) is another matter."), enforcement and monitoring of this limit appear inconsistent.

We now state explicitly in the section on deliberate uranium removal that the lack of uniform adoption of phosphoric acid precipitation or ion-exchange (IX) resin systems suggests that compliance with the shipping limit is not systematically implemented across operations.

In the original version of the manuscript, Figure 4b included a line illustrating the export total assuming universal compliance with the 75 ppm standard, consistent with the reviewer's estimate. We have clarified this line and expanded the caption to explain its significance. Presenting this comparison strengthens the manuscript by placing our scenario-based estimates in regulatory context.

Importantly, even under full compliance with the 75 ppm threshold, annual uranium exports would still exceed 10 tonnes — the quantity above which declaration to the IAEA is required under the Additional Protocol. We now highlight this point explicitly in the Discussion.

Our analysis integrates geological, metallurgical, trade, and process constraints with each step constructed conservatively. While the resulting export magnitudes are substantial, they are consistent with the scale of cobalt production and documented uranium occurrence in the region. We explicitly state the factors that contribute to each estimate, and so readers can see the assumptions and data that underlie our totals.

Guidelines specify a maximum of 70 references. Please confirm with editor if this larger number of references is acceptable.

Response: We have kept all references for now in the interest of keeping the article fully cited for peer review. We are happy to eliminate some references if needed at a later stage.

To conform to conventional practice in science/engineering papers, the following are suggested:

- SI number format should be used throughout the text, except for financial figures. A space is used for the thousand separator for numbers > 9999.

  Response: We have ensured we consistently use SI number format.

- Abbreviations should only be defined if they will be used at least three times in the text. Once an abbreviation is defined, the abbreviation should be used throughout.

  Response: We removed abbreviations that were defined but not reused (e.g., Tenke Fungurume Mine, geometric mean, geometric standard deviation). Remaining abbreviations are used consistently and redefined in captions where necessary.

- It is suggested to use consistent units of kt (or t) in all relevant graphs to make comparison easier.
  Response: We appreciate this point and agree that our conclusions will be clearest with consistent units. We have ensured that all figures now are in terms of tonnes, and mirrored that change in the text.

- Chemical formulae (Methods section) should not be italicised.
  Response: We have changed the formatting so chemical formulae are not italicized. Chemical equations remain italicized.

Reviewer 2

General comments

This manuscript addresses an ambitious, timely, and highly complex issue: the fate of uranium associated with cobalt ores from the Copperbelt of the Democratic Republic of Congo (DRC), and its de facto export through official cobalt supply chains. By bridging economic geology, extractive metallurgy, international trade, nuclear safeguards, and environmental risk, the study tackles a genuine blind spot in current international accounting and regulatory frameworks.

The manuscript presents several noteworthy and original results. Most prominently, the authors quantify, for the first time at this scale, the magnitude of uranium exports embedded in cobalt hydroxide products over a multi-decadal period (2000–2024), combining geological, mineralogical, geochemical, metallurgical, and trade datasets. The integration of mine-level export records with geological mapping and process-based modelling is particularly compelling and represents a major strength of the study. This holistic approach allows the authors to move beyond speculative assessments and provide first-order quantitative constraints on uranium flows that have remained largely unaccounted for.

The work is clearly of high significance to the field and to several related disciplines. For geoscientists and economic geologists, it provides new insights into uranium–cobalt co-mineralization and the downstream behaviour of uranium during hydrometallurgical processing. For the nuclear safeguards and policy communities, it raises important questions regarding undeclared uranium movements within legal supply chains. Compared to the established literature, the manuscript goes well beyond previous descriptive or localized studies by integrating heterogeneous datasets across spatial, temporal, and disciplinary scales. While individual components of the study build on well-established concepts, their synthesis into a coherent, system-level analysis is both original and impactful.
Overall, the data presented convincingly support the main conclusions and claims. The analytical framework is logically constructed, and the progression from geological observations to trade-flow estimates is clear and transparent. The conclusions regarding the scale of uranium export, the limited declaration under international safeguards, and the lack of systematic uranium removal practices are well grounded in the presented evidence. Some interpretations would benefit from additional contextualization—particularly for non-specialist readers—but these points concern clarity and framing rather than the validity of the results.

The data analysis and interpretation are sound. The methods applied to integrate geological, mineralogical, and trade datasets are appropriate and consistent with current standards in the field. No major flaws were identified that would prohibit publication. Any issues raised relate primarily to the need for clearer contextual background, rather than to weaknesses in the analysis itself, and can be addressed through moderate revisions.

The methodology is robust and well suited to the objectives of the study. The authors demonstrate a strong command of both the geological system and the industrial processing chain, and the modelling approach used to estimate uranium carryover is reasonable and clearly justified. The Methods section is generally detailed enough to ensure reproducibility, with clear descriptions of data sources, assumptions, and analytical steps. Minor additions or clarifications in specific places would further enhance transparency but do not require substantive methodological changes.

Given the intrinsic complexity of the system investigated, my main recommendation is to further strengthen and expand the Introduction using well-established and readily accessible contextual information. In particular, a short discussion of the evolution and relative importance of artisanal and informal mining in the DRC Copperbelt over the 2000–2024 period would help contextualize uncertainties in production statistics and material traceability. Likewise, a concise synthesis of existing IAEA findings and concerns regarding uranium in the DRC—especially in relation to cobalt-associated flows—would more explicitly anchor the manuscript within the existing safeguards and regulatory discourse. Brief introductory context on cobalt processing infrastructure in Katanga would also help readers understand where uranium partitioning into cobalt hydroxide is most likely to occur along the value chain.
Finally, translating the quoted uranium quantities into energy-equivalent terms would significantly enhance the broader societal relevance of the results. A short discussion on uranium speciation in heterogenite, particularly at high uranium concentrations, would further strengthen the geochemical interpretation and complement the existing discussion focused on Fe oxyhydroxides.

Response: We sincerely thank the reviewer for this thoughtful and highly constructive assessment of our manuscript. We appreciate the recognition of the study's interdisciplinary scope, methodological rigor, and potential significance for both geoscience and safeguards communities. The suggestions provided are valuable and have helped us strengthen the clarity and contextual framing of the manuscript. We address each point below.

Recommended improvements

I strongly encourage the authors to consider the following additions to the Introduction:

1. Artisanal and informal mining in the DRC (2000–2024)
Since the analysis explicitly covers the period 2000–2024, it would be very useful to include a short discussion of the artisanal and informal mining sector in the DRC Copperbelt. In particular: How has this sector evolved over the considered period? What proportion of total Copperbelt cobalt production does it represent, at least in broad terms? This information would help contextualize uncertainties related to production statistics, traceability, and undeclared material flows.

Response: We agree that artisanal and informal mining is an important contextual factor, particularly given the temporal scope of our analysis (2000–2024) and its implications for production statistics and traceability.

Because the Introduction already bridges multiple disciplinary domains, we were concerned that adding a detailed discussion at that stage would reduce clarity and dilute the manuscript's central framing. Instead, we have added a dedicated subsection entitled *"A note on artisanal mining and uranium grades"* following the presentation of our total uranium export estimates.
This addition provides the context requested by the reviewer while preserving the clarity and structure of the Introduction.

2. IAEA findings on uranium in the DRC and cobalt-associated flows
A short synthesis of the main conclusions and concerns raised by the IAEA regarding uranium in the DRC would be highly valuable. In particular: What is currently known and officially reported about uranium trafficking or undeclared production? What has been reported specifically in relation to cobalt-associated uranium during the 2000–2024 period? This would anchor the manuscript more explicitly within the existing safeguards and regulatory discourse.

Response: We agree that more explicitly situating the manuscript within existing IAEA reporting and safeguards information strengthens its impact.

In the revised manuscript, we clarify that the Democratic Republic of Congo reports no uranium exports to the International Atomic Energy Agency (IAEA) during the study period. The principal global reference on uranium production, the 2024 IAEA *Red Book*, does not identify cobalt production in the DRC as a uranium source.

We also cite publicly available documentation from the Finnish nuclear safeguards authority describing uranium detected in cobalt shipments from the DRC to Kokkola, its subsequent separation, and placement under safeguards. To our knowledge, this remains the only publicly documented instance of uranium originating from the DRC cobalt supply chain that was placed under safeguards.

We have refined the Introduction to make clear that this represents the current extent of public knowledge on this issue.

3. Cobalt processing infrastructure in Katanga
The manuscript would benefit from 2–3 introductory lines describing the processing units and refineries that are actually capable of treating cobalt ores in the Katanga region. This would help readers better understand where and how uranium partitioning into cobalt hydroxide is likely to occur along the value chain.

Response: We agree that brief clarification of the processing infrastructure in Katanga helps readers understand where uranium partitioning into cobalt hydroxide is most likely to occur.

To preserve the clarity of the Introduction, we maintain the existing structure in which the general uranium co-occurrence issue is introduced first, followed by more detailed discussion of hydrometallurgical processing pathways in the subsequent sections. We believe this sequencing improves logical flow, as readers are first introduced to the problem and then guided through the relevant processing mechanisms.

However, we have added additional clarifying language in the processing section (mostly in response to comments from Reviewer 1) to more clearly describe where uranium partitioning into cobalt hydroxide occurs along the value chain. In combination with the new subsection addressing artisanal mining, this improves the manuscript without overloading the Introduction.

4. Energy-equivalent context for uranium quantities
Given the quantities quoted in lines 16–17, it would be extremely useful to briefly translate these figures into energy-equivalent terms, i.e.: What do these uranium masses correspond to in terms of potential electricity generation or nuclear fuel use? This would greatly enhance the broader societal relevance and impact of the results.

Response: We very much agree with this comment and recognize that tonnes of uranium may not mean as much to a broad readership compared to amounts of energy or numbers of warheads. To add this context, we now point to three conversions in our discussion:

- 200 tonnes of natural uranium are required to generate 1GW of electricity for one year in a standard light water reactor (assuming 4.5% enriched fuel).

- 3.3 tonnes of natural uranium are required to make a nuclear weapon (15kg of uranium enriched at 93% in the 235 isotope).
- 1 tonne of uranium costs $201,000 on the open market

We use these first-order estimations as they are accurate, and do not require us to assume specific end uses. They simply serve the purposes indicated by this reviewer of increasing societal relevance.

5. Uranium speciation in heterogenite
Finally, I recommend adding a short discussion on the speciation of uranium in heterogenite, particularly at high U concentrations. Specifically: Is uranium considered to be primarily adsorbed, and if so, in the form of which complexes? Is co-precipitation invoked for the highest U contents? Adsorption is discussed on page 11, but the focus there is mainly on Fe oxy-hydroxides; extending this discussion to heterogenite would be highly valuable.

Response: We have expanded the discussion of uranium occurrence in heterogenite to provide greater geochemical specificity.

We now clarify that adsorption of uranyl species onto heterogenite surfaces is considered the most likely mechanism, even at relatively high uranium concentrations. We also acknowledge that the literature is not definitive and that lattice substitution of uranyl species or inclusion of discrete uranium oxide particles remain possible under certain conditions.

Importantly, regardless of the precise structural relationship, uranium associated with heterogenite is readily mobilized under the acidic conditions used in cobalt hydrometallurgical processing. Thus, the exact speciation does not materially affect uranium dissolution behavior during processing.

The later discussion of adsorption onto Fe oxyhydroxides concerns a distinct mechanism related to incidental uranium removal and is therefore treated separately.

Minor and Specific Comments

Lines 9–10: Please indicate what fraction of global reserves this corresponds to.

Response: We have adjusted the sentence to say that over 70% of global cobalt supply is sourced from the DRC.

Line 15: It would also be helpful to specify the percentage here.

Response: We have adjusted the sentence to say that over 95% of cobalt is exported from the DRC as crude hydroxide.

Line 42: “an oxyhydroxyde” → an oxyhydroxide.

Response: Changed.

Line 43: Please specify the quantity more precisely.

Response: We have updated the sentence to state that Uranium concentrations may be up to 3% by weight in heterogenite samples.

Line 74: One reason for highly U-enriched heterogenite is that, in the primary (sulfide) mineralization, uraninite is frequently associated with Co–Ni–Cu sulfides in deposits known for overall U enrichment. This point could be explicitly stated.

Response: We prefer not to make this detailed geologic point in this section that relates more closely to metallurgy. We clarified the geological elemental associations in the 'Modeling uranium prevalence in cobalt ores' subsection to address this point along with similar feedback from Reviewer 3.

Line 102: Suggested rephrasing: "They occur in the same mineral only in the oxidized ore."

Response: We have added the specification that the co-occurrence is in oxidized ores.

Reviewer 3

This is an interesting and sound paper, which focuses on a little-studied theme, relevant for both pollution, public health and nuclear safeguards.

Response: We thank the reviewer for their careful reading of the manuscript and for their thoughtful and constructive geological insights. We address the specific points below.

I don't have a lot of comments, safe for the geologic presence of uranium in the Copperbelt. I fully agree that fault zones may contribute, since the mineralized rocks are exposed, to the migration of uranium. But the role of fault zones is more complex than sketched in the paper. U primary stock in the sedimentary basin is geologically "old" (in the order of 800 Myr, roughly contemporaneous with the Cu-Co deposit, Decrée et al., 2014) and fault zones contributed to multiphase migration of this metal along the 800 Myr-long geological history, and notably during the Lufilian orogeny (Decrée et al., 2011). Intrinsic U mobility in oxidizing conditions, prevailing since about 80 Myr in the study area (De Putter and Ruffet, 2020), has allowed its migration and coprecipitation with oxidized cobalt ore (heterogenite) – a process likely enhanced in the last 10 Myr, as rightly stated in the paper. This is just to emphasize that there are Neoproterozoic fault-related subvertical "primary" U deposits (such as Shinkolobwe), comprising a surprisingly complex metal paragenesis: Au + U + Se + Mo + Ni + PGE, and more. Whether such a deposit is at the origin of all the other showings in Katanga, through migration and dissemination of this "primary" U stock is still open for debate. This geological nitpicking doesn't affect the assumption that recent exposure of the deposits to oxidizing conditions have fostered U lateral migration, either from Shinkolobwe-type deposit(s) or from other "secondary" reconcentrated stocks of U along the last 800 Myr.

Response: We are pleased that a reviewer so familiar with the local geology agrees in general with our geologic approach, and we appreciate the guidance for clarifying these additional complexities related to uranium concentration and mobilization.

We have distilled the important points made by the reviewer into the main text. The opening lines of the **Higher uranium grades along faults** subsection have been slightly expanded to clarify that fault-bound uranium deposits (such as Shinkolobwe) formed in the Neoproterozoic-to-Cambrian due to tectonic activity, and then oxidative meteoric weathering (starting 80 Ma and intensifying since 10-5 Ma) mobilized this uranium and redeposited it along with cobalt minerals. We have included the De Putter and Ruffet, 2020 reference for the onset of oxidative weathering.

We agree with the reviewer that these geological refinements do not affect the core assumption underpinning our modeling—namely, that recent exposure of deposits to oxidizing conditions has promoted lateral uranium migration and association with oxidized cobalt ores.

In this context, I would turn the sentence "cobalt ores co-occur with uranium" (line 244) the other way: "uranium co-occurs with oxidized cobalt ore".

Response: We changed the word order of the sentence as suggested by the reviewer, as well as a similar sentence in the abstract.

The sentence "DRC copper deposits commonly contain economic grades of cobalt" (line 37) makes me feel uncomfortable. The Copperbelt hosts a unique type of copper and cobalt deposit, the only one on Earth where cobalt is enriched to the point that it is a commodity exploited on its own. In other deposits, it is generally a by-product of another metal (typically in ultramafic Ni-Co deposits).

Response: We agree with this reviewer that we mischaracterized the nature of deposits with this phrasing. While editing the introduction to incorporate all reviewer feedback, we noted that it was no longer necessary to bring up relative grades of copper and cobalt in this sentence, and so we have replaced the clause indicated above with "Recently,".

In a broader way, the sentence "cobalt's rise via the copper boom" (line 239) is not fully correct; there were copper booms without a cobalt rise in the past. The present cobalt rise is mostly due to specific needs in this metal since WWII (De Putter 2019, no need to cite!) even if, in some instances, end-products (as e-powered cars) require both metals.

Response: We are thankful for this reviewer's continued help in clarifying the nature of the DRC cobalt industry. We have discarded the language indicated above, and instead say: "This secondhand uranium production mirrors the byproduct nature by which cobalt is recovered from some copper operations".

Line 33 and fig. 1: I don't like "bands" of Roan sediments (or of metalliferous rocks): "outcrops" would be much better.

Response: We have changed the wording from "bands" to "outcrops" in both instances.

Last detail: I didn't check if Shinko was closed "80 years ago" (line 26) … I would have said later but I may be wrong.

Response: The sentence in question refers to the fact that uranium from the DRC was first used in nuclear weapons approximately 80 years ago; it does not state that the mine itself was closed 80 years ago. We have lightly adjusted the phrasing to ensure clarity.
